# Supplementary material for: A universal approach for the synthesis of two-dimensional binary compounds
Source: Nat Commun. 2019 Jul 4;10:2957. doi: 10.1038/s41467-019-11075-2 (PMC6609655; doi:10.1038/s41467-019-11075-2)
Supplement: Supplementary file 1 — Supplementary Information [file 41467_2019_11075_MOESM1_ESM.pdf]

Supplementary Information

A universal approach for the synthesis of two-dimensional binary compounds

Shivayogimath et al.

Supplementary Methods

Sample characterisation. Optical microscopy images were taken in a Nikon Eclipse L200N optical microscope. Scanning electron microscopy (SEM) was conducted in a Zeiss Supra 40VP operated in in-lens detection mode at 5.00 keV. Atomic force microscopy (AFM) scans were done in a Bruker AFM Dimension Icon. X-ray photoelectron spectroscopy (XPS) measurements were conducted in a Thermo Fisher Scientific K-alpha XPS system using an AlKα X-ray source (1486.7 eV). Acquisition parameters were kept constant for all survey and high-resolution scans (300 ms x 10 scans). Raman spectroscopy was conducted in a Thermo Fisher DXR microscope equipped with a 455 nm laser (5 mW, 10 s x 5 exposure time, 50x objective). Photoluminescence spectra were obtained using a custom spectroscopy setup built from a Nikon Eclipse Ti-U inverted microscope and equipped with a 407 nm diode laser and a Shamrock 303i spectrometer with a 450 nm longpass filtrer (30 µW, 1 s x 5 exposure time, 100x objective, 0.9 NA).

Electron backscatter diffraction (EBSD). EBSD measurements were used to determine the surface grain orientation of Au-*M* alloys after processing, which in the case of Supplementary Figure 1 was an Au-V alloy. Measurements were done in a FEI Nova NANO SEM 600 using the following parameters: 15 kV, 12 nA, 10.5 mm working distance, 50 µm aperture, a 10 µm x 10 µm scan area and a step size of 280 nm. The obtained diffraction patterns were indexed against pure Au and Au-V (85%-15%) structures, and were found to match with the pure Au phase only. Various different regions were also scanned to ensure homogeneity throughout the sample. The SEM image in Supplementary Figure 1 was taken in the same instrument, using a secondary electron detector.

Transmission electron microscopy (TEM). TEM characterisation of W, Mo chalcogenides and CoS_2 was done in an FEI Tecnai T20 G2 operated at 200 kV. Bright field HRTEM images of transferred VN films were taken in an FEI Titan 80-300 environmental TEM with post-specimen spherical aberration correction, operated at 80 kV.

Terahertz-time domain spectroscopy (THz-TDS). THz-TDS mapping of the sheet conductivity of VN on quartz was acquired using a Picometrix T-ray 4000 fibre-coupled spectrometer. The sample was raster-scanned to form a spatial map with 200 µm step size and a resolution of ≈ 350 µm at 1 THz. The frequency-dependent sheet conductivity was determined as described in detail in previous reports[1,2].

Ellipsometry. Ellipsometry measurements were performed in reflection mode with a rotating compensator spectroscopic ellipsometer (M-2000, J.A. Woollam Co.). The measurement of the ellipsometric quantities ψ and Δ in the spectral range 0.7-5.9 eV was repeated at seven angles of incidence (45-75°) at each measurement spot using a collimated beam with a spot size of approximately 200 x 300 µm. Four spots were measured on the transferred VN film on quartz. The multi-angle ψ and Δ spectra for each spot were fitted simultaneously to an optical model, with thickness and optical functions of

VN being the unknown parameters. The optical functions of the quartz substrate were determined in a separate ellipsometry measurement. A multi-sample analysis routine was employed to accurately determine both the thickness and the optical functions of VN avoiding the risk of cross-correlation between fitted parameters. In one of the four spots, the thickness was fixed to the value of 5.5 nm as determined by AFM. The thicknesses of the three remaining spots were fitted independently. The optical functions of VN were parameterised as explained in the caption of Supplementary Figure 21, but were not allowed to vary from spot to spot.

Density-functional theory (DFT) calculations. We use first-principles calculations based on density-functional theory to obtain the electronic properties of select chalcogenides. We employ a first-principles approach based on Kohn-Sham density functional theory (KS-DFT)[3], as implemented in the Quantum ESPRESSO code[4]. The exchange correlation energy is described by the generalized gradient approximation (GGA) using the PBE[5] functional. Interactions between valence and core electrons are described by Troullier-Martins pseudopotentials[6].

Supplementary Table 1. Supplementary device performance characteristics of measured MoS_2 devices.

Device Number	μ (± 5 cm^2/Vs)*	On-off ratio**	Hysteresis (± 2V)
1	4	10^3	26
2	20	10^4	9
3	25	10^4	16
4	30	10^4	11
5	30	10^4	7
6	30	10^2	7
7	5	10^3	11
8	5	10^3	12
9	15	10^5	34
10	15	10^4	14

*: Uncertainty in the estimation of μ for these devices is a result of the device geometry due to transfer of MoS_2 regions to pre-patterned electrodes (see main text).

**: Gate leakage prevented driving the devices to saturation – the on-off ratio here is calculated from the largest achieved ‘on’ current in each case.

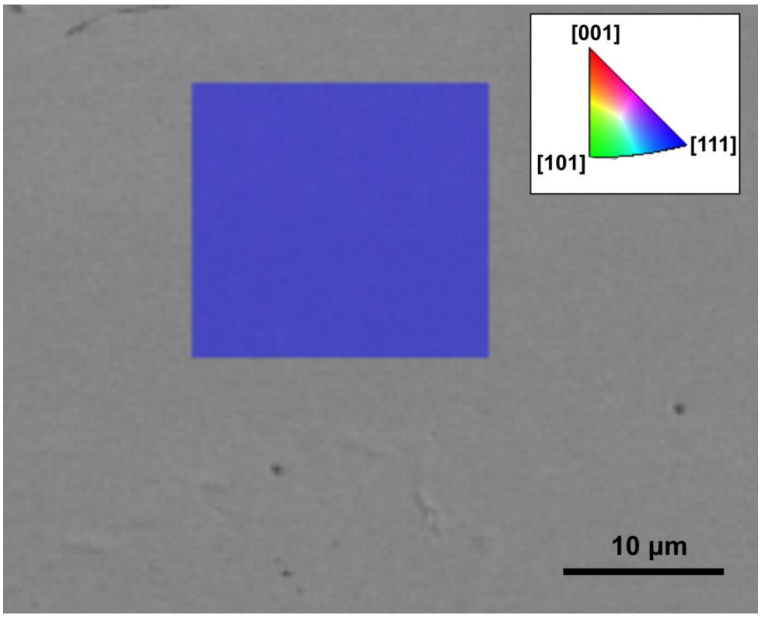

Supplementary Figure 1. EBSD of post-process Au alloys on c-plane sapphire. EBSD map of a ~100 μm^2 area overlaid onto an SEM image of the alloy surface. The EBSD clearly shows that the surface has the Au {111} orientation.

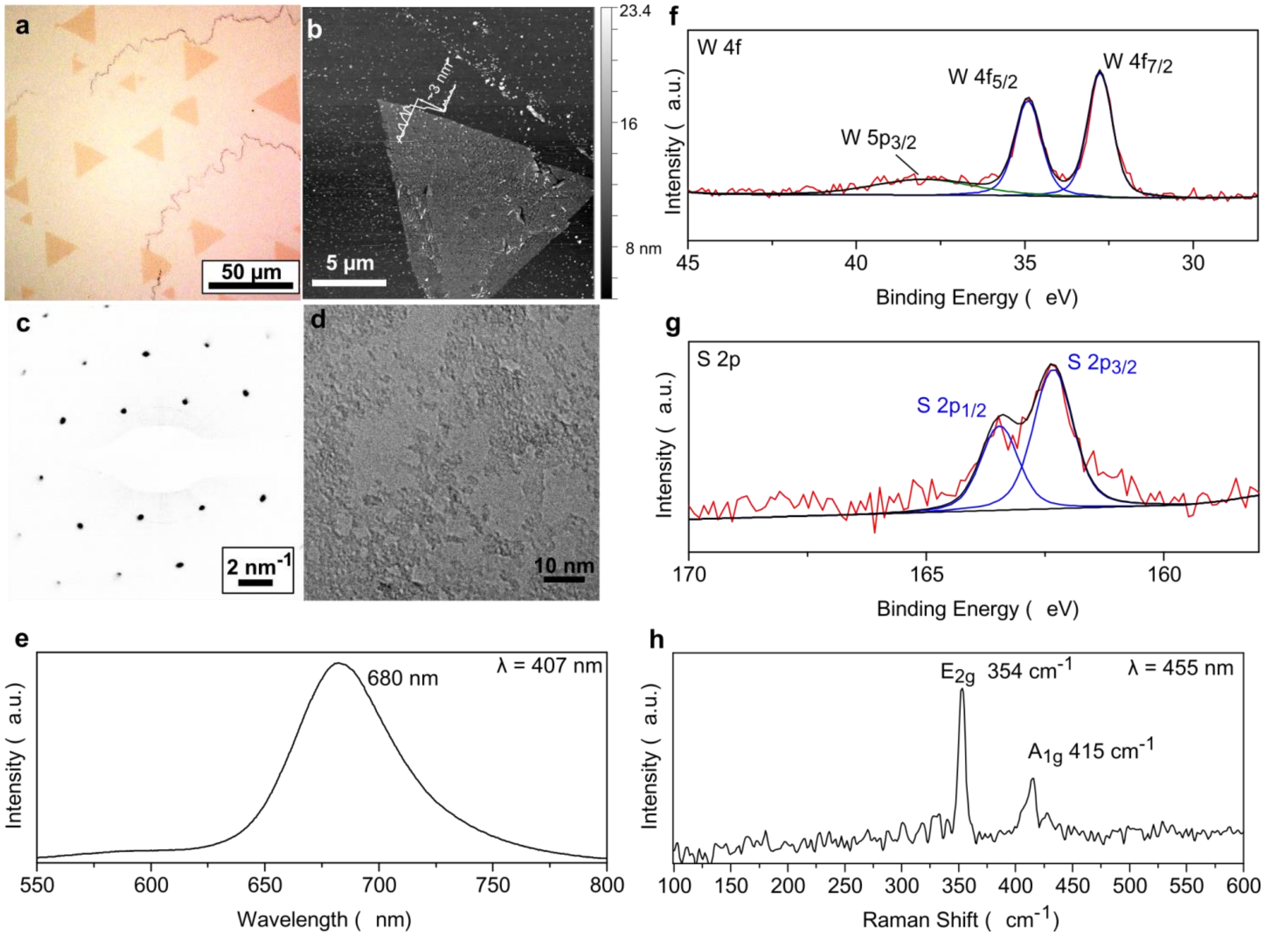

Supplementary Figure 2. Characterisation of tungsten disulphide (WS_2). (a) Optical image of WS_2 domains on gold, showing evidence of epitaxial alignment with the underlying substrate. **(b)** Atomic force microscopy (AFM) image of a WS_2 domain transferred onto 90 nm SiO_2 on Si substrate; the measured thickness of the domain is ~ 3 nm, which is within the thickness of a monolayer when taking into account polymer residues, surface oxidation, and tip and substrate interactions[7,8]. **(c)** Selected area diffraction (SAED) pattern and **(d)** bright field TEM of a WS_2 domain transferred onto TEM grids. **(e)** Photoluminescence spectrum of WS_2 domains transferred onto 90 nm SiO_2/Si substrates, showing a strong peak at 680 nm. The peak is redshifted from reported values for monolayer WS_2[9], which we suspect is due to screening and doping effects from transfer residues. **(f-g)** High resolution XPS spectra of **(f)** W 4f and **(g)** S 2p regions of as-grown WS_2 on the gold surface, where the peaks at 32.8 eV (W $4f_{7/2}$), 34.9 eV (W $4f_{5/2}$), 162.3 eV (S $2p_{3/2}$) and 163.5 eV (S $2p_{1/2}$) correspond to WS_2 (NIST XPS database). **(h)** Raman spectra of WS_2 domains on gold, indicating monolayer thickness[10].

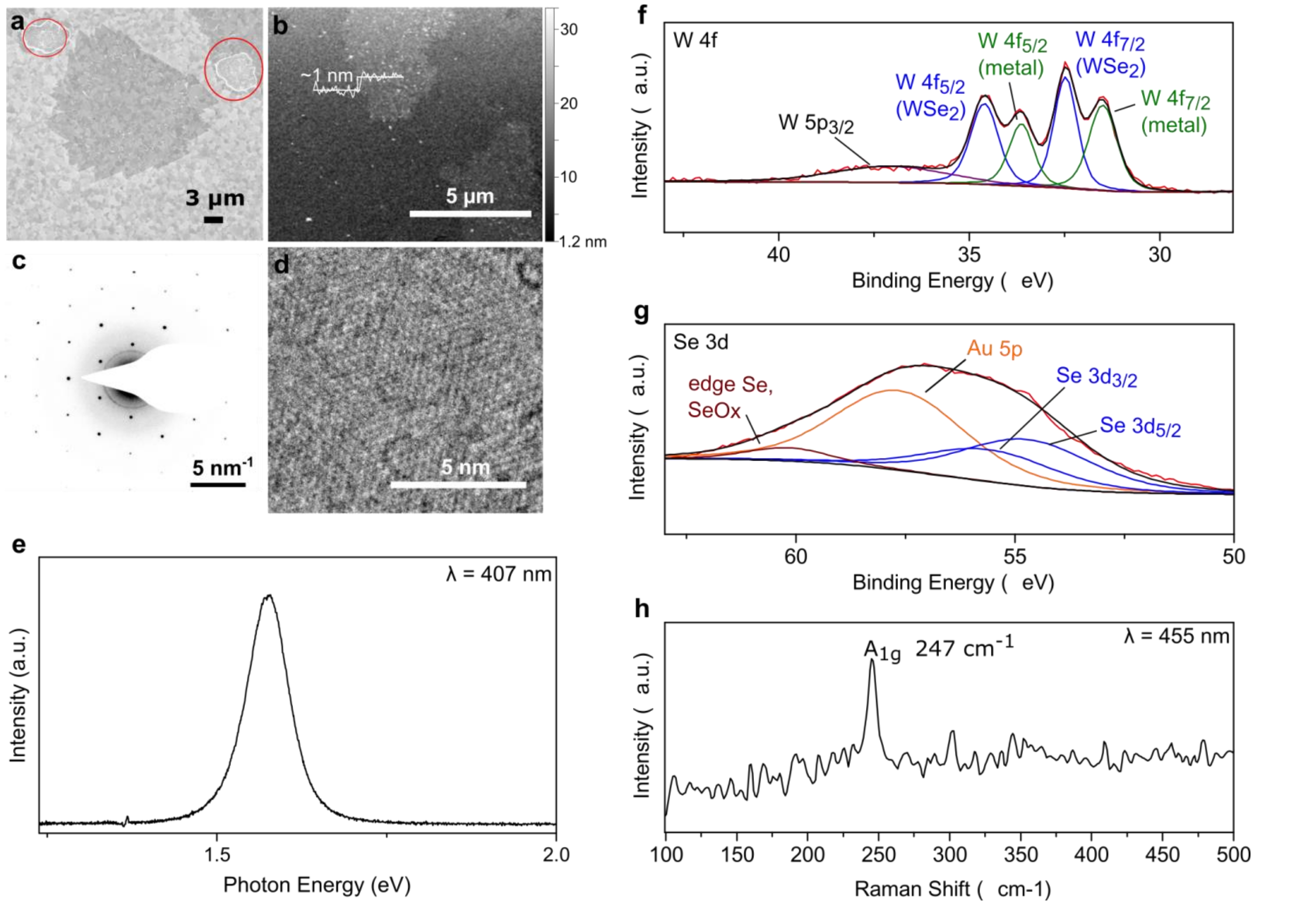

Supplementary Figure 3. Characterisation of tungsten diselenide (WSe_2). (a) SEM image of WSe_2 domains on gold. Red circles in **(a)** indicate areas where the gold film has dewetted the underlying tungsten layer due to its thickness (initial thickness of the gold film here was 300 nm). **(b)** AFM image of a WSe_2 domain transferred onto 90 nm SiO_2/Si substrate; the measured thickness of the domain is ~ 1 nm, not accounting for transfer residues and chemical contrast between tip and substrate. **(c)** SAED pattern and **(d)** bright field TEM of WSe_2 transferred onto TEM grids. **(e)** Photoluminescence spectrum of WSe_2 domains transferred onto 90 nm SiO_2/Si substrates, which shows a strong peak at 1.57 eV. **(f-g)** High resolution XPS spectra of **(f)** W 4f and **(g)** Se 3d regions of as-grown WSe_2 on the gold surface. Peaks at 32.5 eV and 34.6 eV in (f) correspond to WSe_2, while the peaks at 31.5 eV and 33.6 eV correspond to the exposed W metal in the de-wetted areas in (a) (NIST XPS Database). Peaks in (g) at 54.7 eV and 55.6 eV correspond to Se 3d peaks of WSe_2, while we attribute the peak at 60.2 eV to oxidised/edge selenides[11,12]. **(h)** Raman spectra of WSe_2 domains on gold, which indicates that they are monolayers[13].

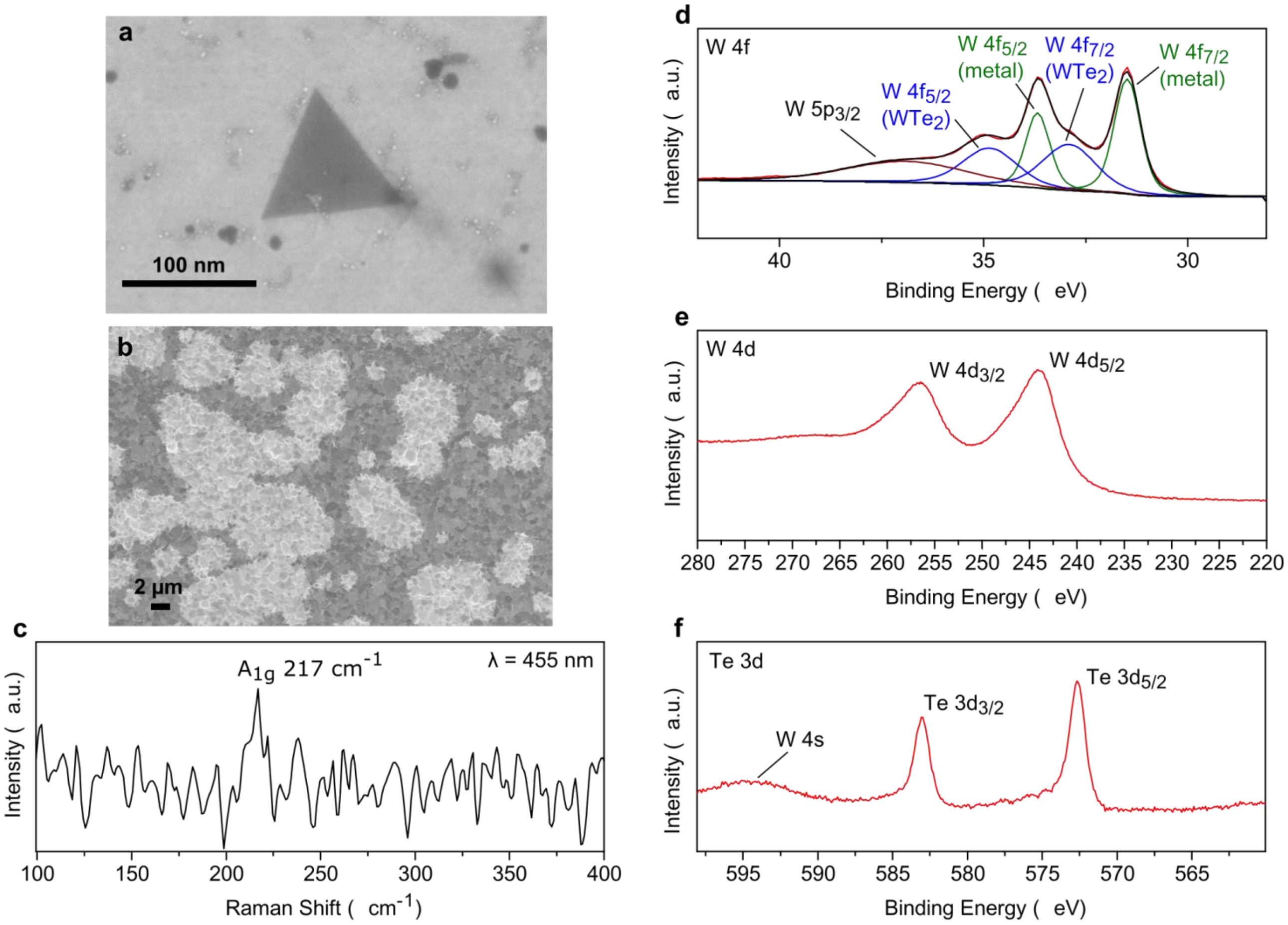

Supplementary Figure 4. Characterisation of tungsten ditelluride (WTe_2). (a) High magnification of isolated WTe_2 domains and **(b)** low magnification SEM images of merged WTe_2 domains on gold. Dark coloured regions in **(b)** are partially grown WTe_2 films. **(c)** Raman spectra of WTe_2 on gold, which indicates that the film is a monolayer[14], **(d-f)** High resolution XPS spectra of **(d)** W 4f, **(e)** W 4d and **(f)** Te 3d regions of as-grown WTe_2 on gold. W 4f peaks in (d) at 32.9 eV and 34.85 eV correspond to WTe_2, whereas the peaks at 31.5 eV and 33.7 eV correspond to W metal from dewetted areas, similar to the case of WSe_2 (the initial gold film thickness was 300 nm in this case as well). W 4d peaks in (e) at 244.2 eV and 256.5 eV correspond to reported values for WTe_2[15]. Te 3d peaks in (f) are at 572.7 eV and 583 eV, corresponding to WTe_2[15]. WTe_2 films did not appear to survive transfer onto 90 nm SiO_2 on Si substrates.

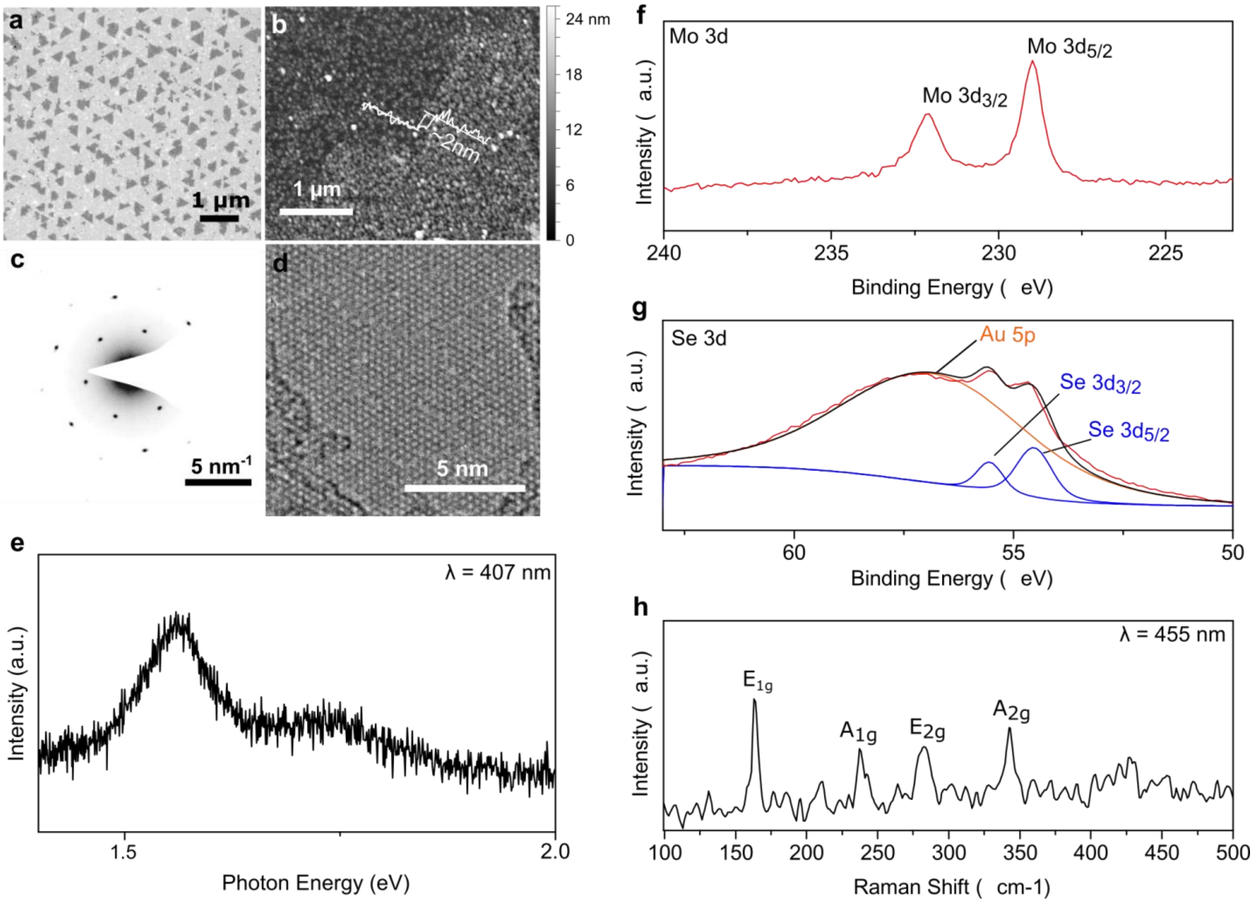

Supplementary Figure 5. Characterisation of molybdenum diselenide ($MoSe_2$). (a) SEM image of $MoSe_2$ domains on gold, showing evidence of epitaxial alignment with the underlying gold. **(b)** AFM on $MoSe_2$ domains transferred onto 90 nm SiO_2/Si substrate; the measured thickness of the domain is ~ 2 nm, not accounting for transfer residues and chemical contrast between tip and substrate. **(c)** SAED pattern and **(d)** high resolution TEM image of $MoSe_2$ domains transferred onto TEM grids. Visible defects are caused by knock-on damage during imaging at 200 kV. **(e)** Photoluminescence spectrum of $MoSe_2$ domains transferred onto 90 nm SiO_2/Si substrates, showing A and B exciton peaks at 1.57 eV and 1.75 eV, respectively[16]. **(f-g)** High resolution XPS spectra of the **(f**) Mo 3d and **(g)** S 2p regions of $MoSe_2$ on gold. Mo 3d peaks in (f) at 228.9 eV and 232.1 eV and Se 3d peaks in (g) at 54.6 eV and 55.5 eV correspond to $MoSe_2$[17]. **(h)** Raman spectra of $MoSe_2$ transferred onto 90 nm SiO_2/Si substrate; the pronounced E_{1g}, E_{2g}, and A_{2g} peaks are attributed to the excitation wavelength used in this work[18].

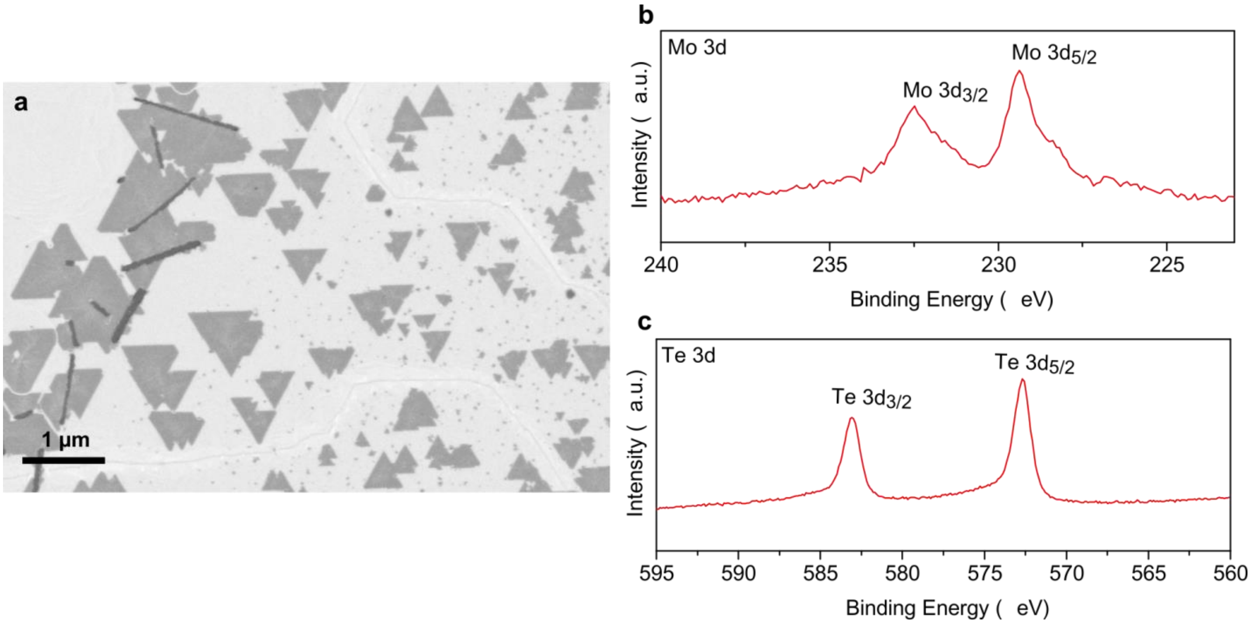

Supplementary Figure 6. Characterisation of molybdenum ditelluride ($MoTe_2$). (a) SEM image of a $MoTe_2$ domain on gold. **(b-c)** High resolution XPS spectra of the **(b)** Mo 3d and **(c)** Te 3d regions of $MoTe_2$ on gold. Mo 3d peaks in (b) at 229.2 eV and 232.3 eV and Te 3d peaks in (c) at 572.6 eV and 582.9 eV correspond to $MoTe_2$[19]. We were unable to detect a sufficiently strong Raman signal from the domains on gold with our experimental setup, and the domains did not appear to survive transfer onto SiO_2 substrates.

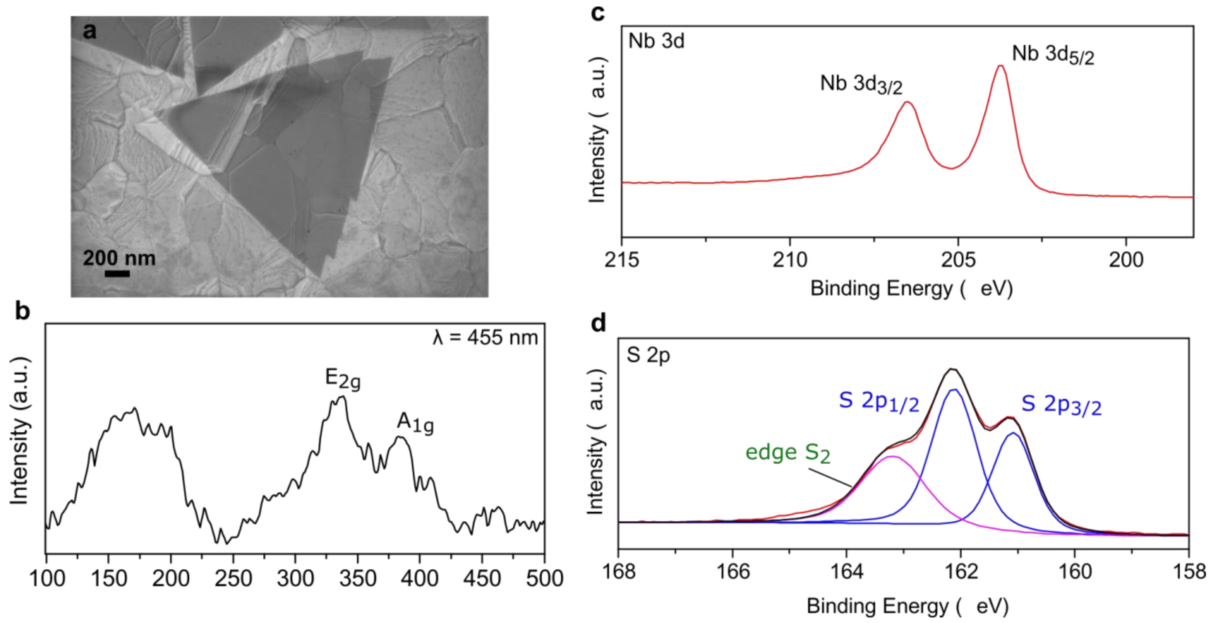

Supplementary Figure 7. Characterisation of niobium disulphide (NbS_2). (a) SEM image of an NbS_2 domain on gold. (b) Raman spectrum of an NbS_2 film on gold, where the E_{2g} and A_{1g} correspond to reported values for NbS_2[20]. High resolution XPS spectra of the **(c)** Nb 3d and **(d)** S 2p regions of NbS_2 on gold. Nb 3d peaks in (c) are at 203.7 eV and 206.5 eV and S 2p peaks in (d) are at 161.1 eV, 162.2 eV and 163.2 eV.

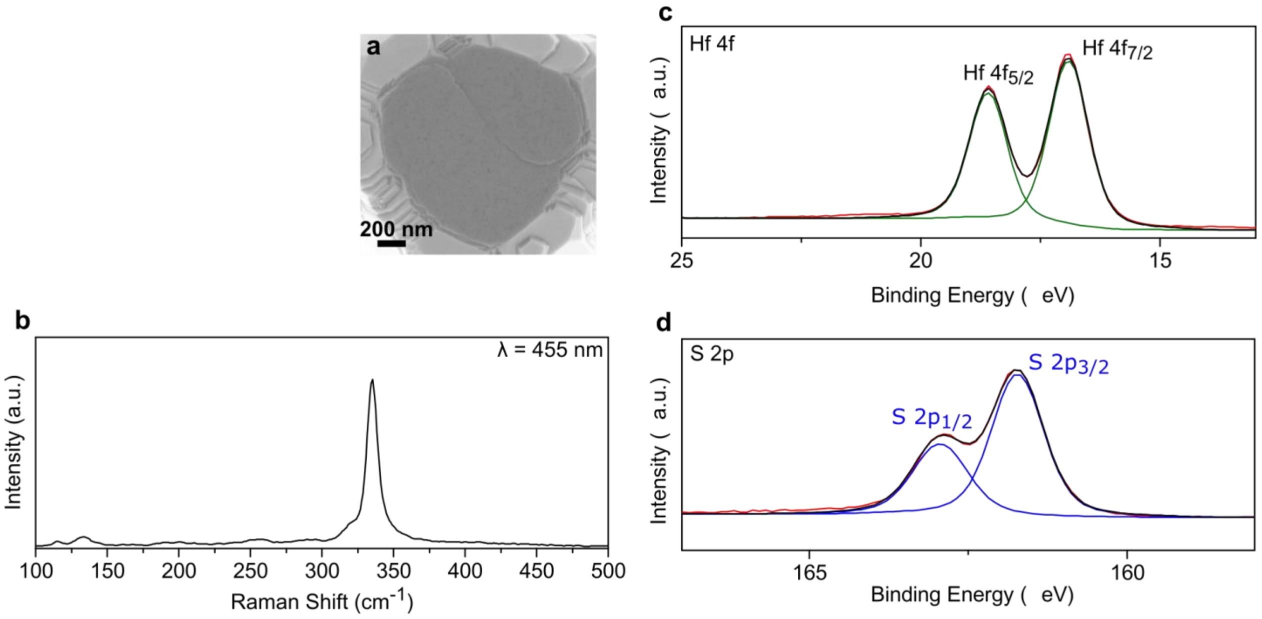

Supplementary Figure 8. Characterisation of hafnium disulphide (HfS_2). (a) High magnification SEM image of an isolated HfS_2 domain on gold. **(b)** Raman spectrum of an HfS_2 film on gold, which shows a pronounced peak at ~335 cm^{-1} corresponding to reported values for HfS_2[21]. **(c-d)** High resolution XPS spectra of the **(c**) Hf 4f and **(d)** S 2p regions of HfS_2 on gold; Hf 4f peaks are at 16.9 eV and 18.6 eV and S 2p peaks are at 161.7 eV and 162.9 eV. The Hf:S ratio here is 1:2.

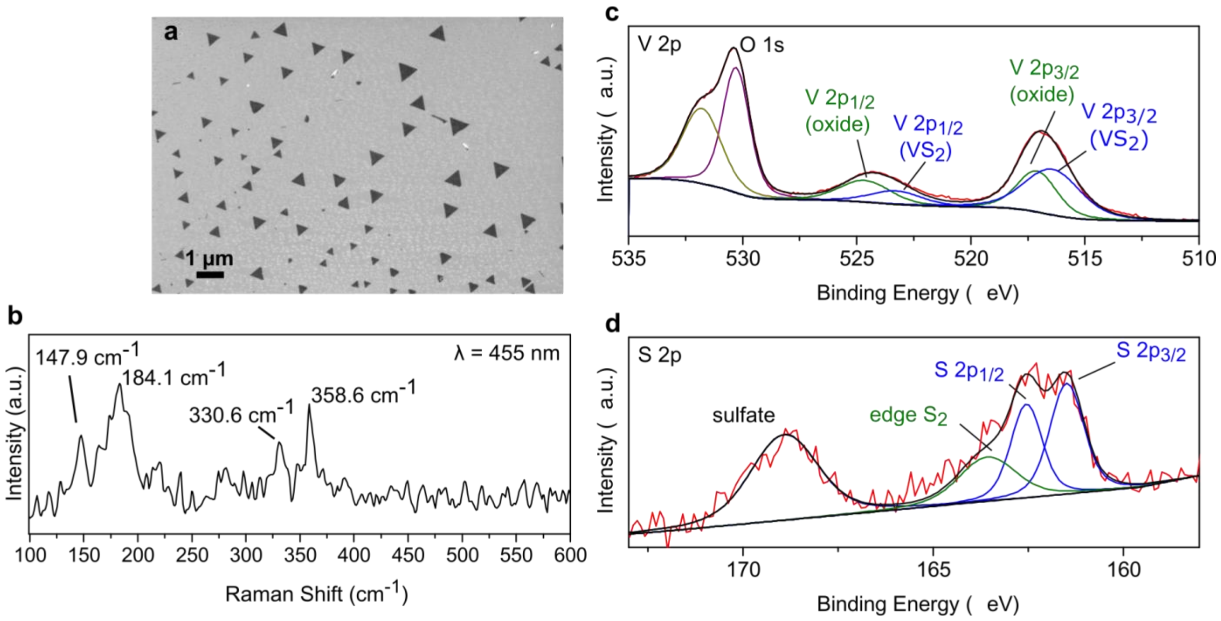

Supplementary Figure 9. Characterisation of vanadium disulphide (VS_2). (a) SEM images of VS_2 domains on gold, showing evidence of epitaxial alignment with the underlying Au (111) substrate. **(b)** Raman spectrum of VS_2 film on gold, where the peaks correspond to reported values for VS_2 nanosheets[22]. **(c-d)** XPS spectra of the gold surface post-growth: **(c)** V 2p and **(d)** S 2p scan. The V 2p spectrum shows peaks at 516.4 eV and 523.3 eV, which correspond to the V^{4+} oxidation state in VS_2[23]. The peaks at 517.1 eV and 524.7 eV can be attributed to vanadium oxides[11]. The S 2p peaks at 161.5 eV and 162.6 eV are attributed to VS_2. VS_2 is air-sensitive and degrades upon exposure to ambient, as evidenced by the prominent sulphate peak in (d).

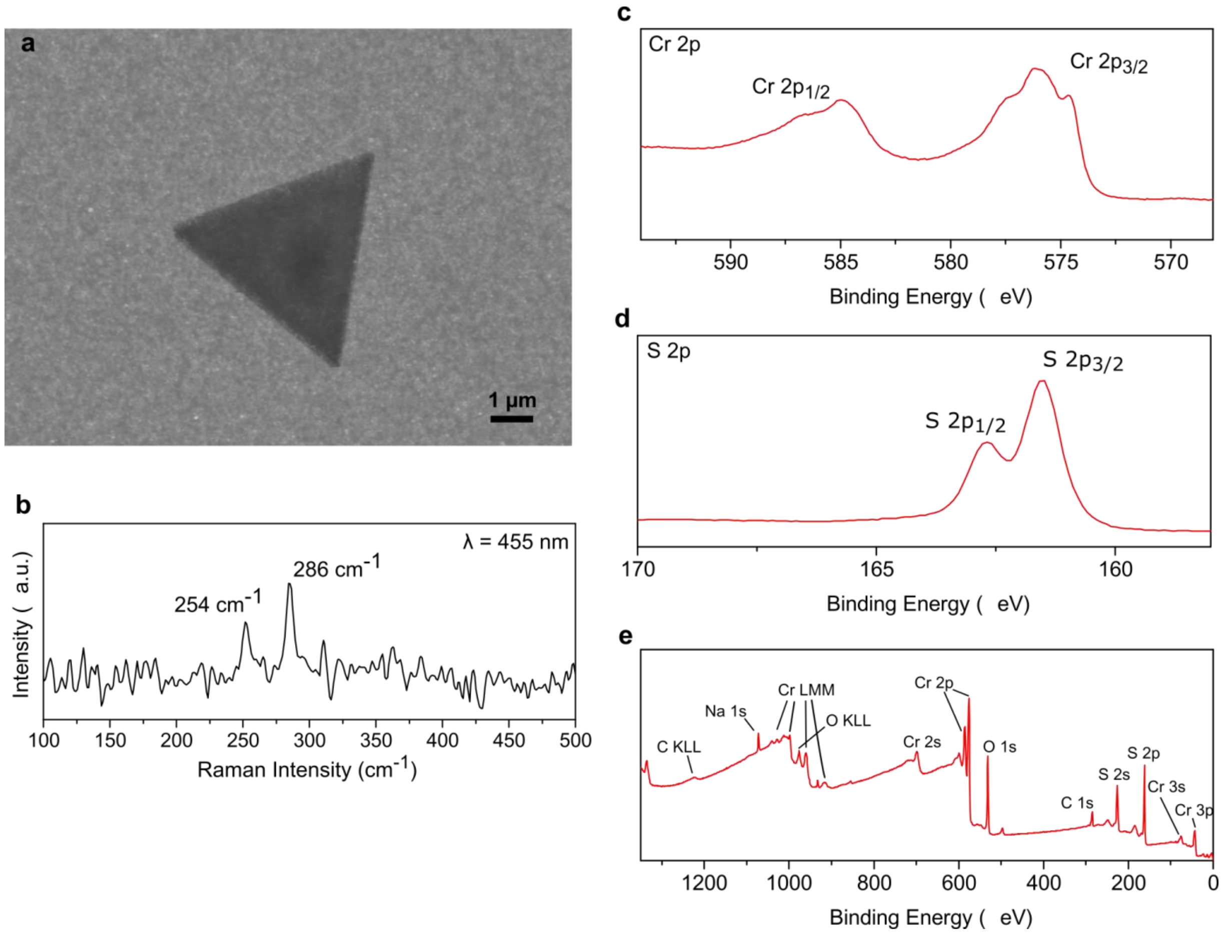

Supplementary Figure 10. Characterisation of chromium disulphide (CrS_2). (a) SEM image of a CrS_2 domain on gold. **(b)** Raman spectrum of a CrS_2 domain on gold. **(c-e)** XPS spectra of the gold surface post-growth: **(c)** Cr 2p, **(d)** S 2p, and **(e)** survey scan.

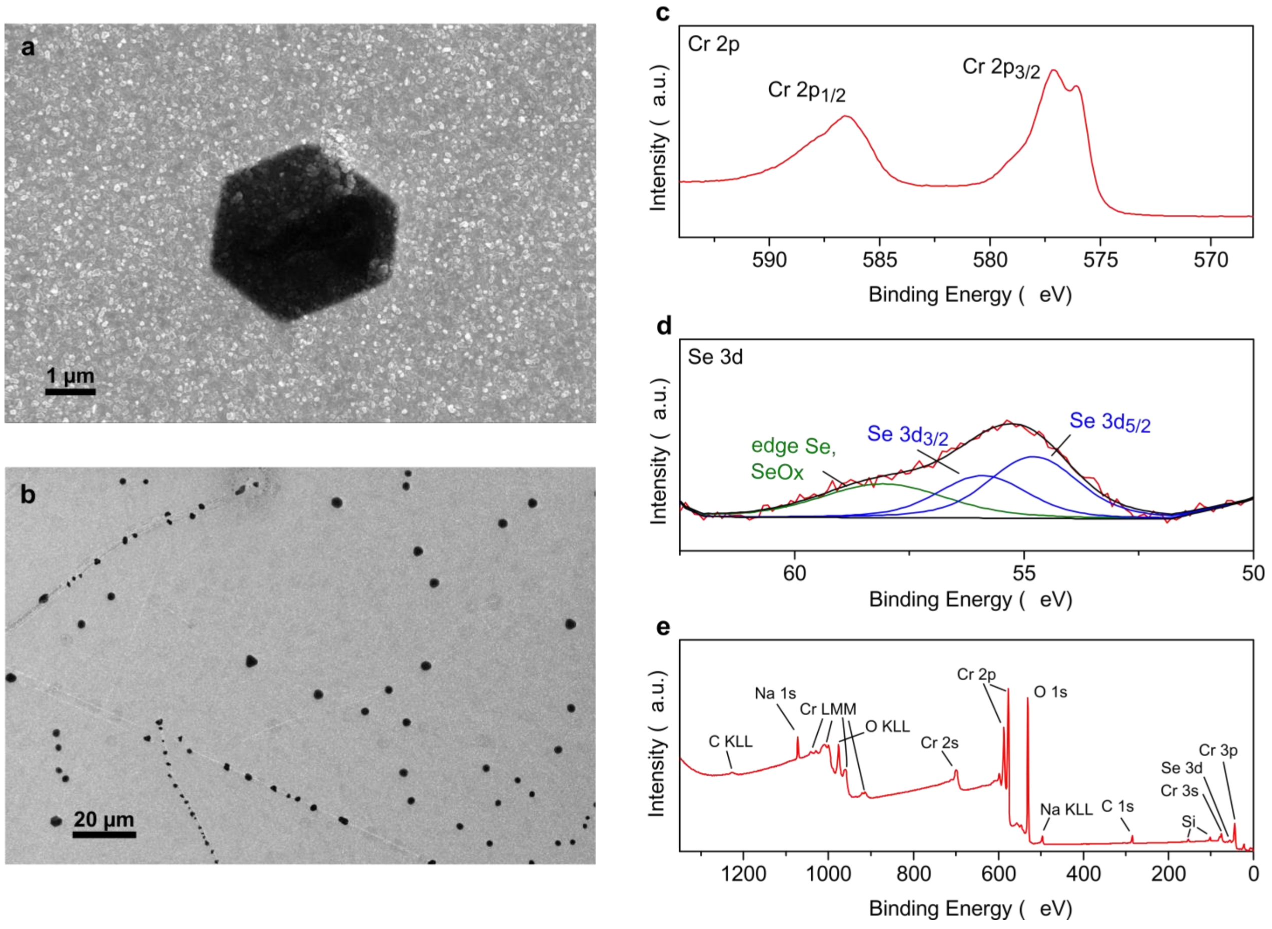

Supplementary Figure 11. Characterisation of chromium diselenide ($CrSe_2$). (a) High magnification and **(b)** low magnification SEM images of $CrSe_2$ on gold. **(c-e)** XPS spectra of the gold surface post-growth: **(c)** Cr 2p, **(d)** Se 3d, and **(e)** survey scan.

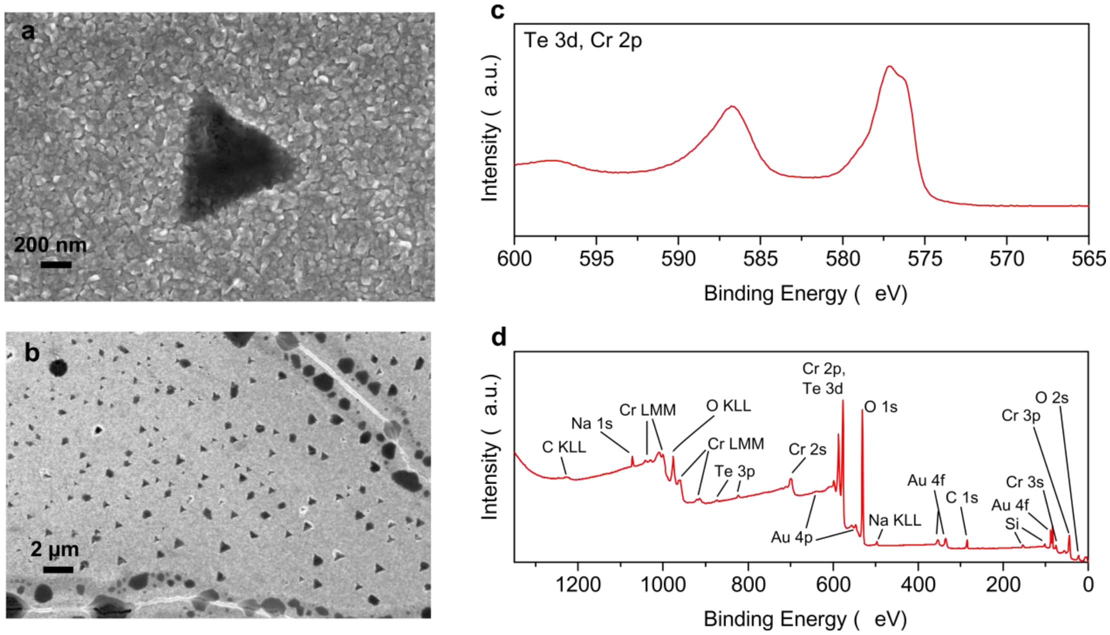

Supplementary Figure 12. Characterisation of chromium ditelluride ($CrTe_2$). (a) High magnification and **(b)** low magnification SEM images of $CrTe_2$ on gold. **(c-d)** XPS spectra of the gold surface post-growth: **(c)** Te 3d,Cr 2p and **(d)** survey scan.

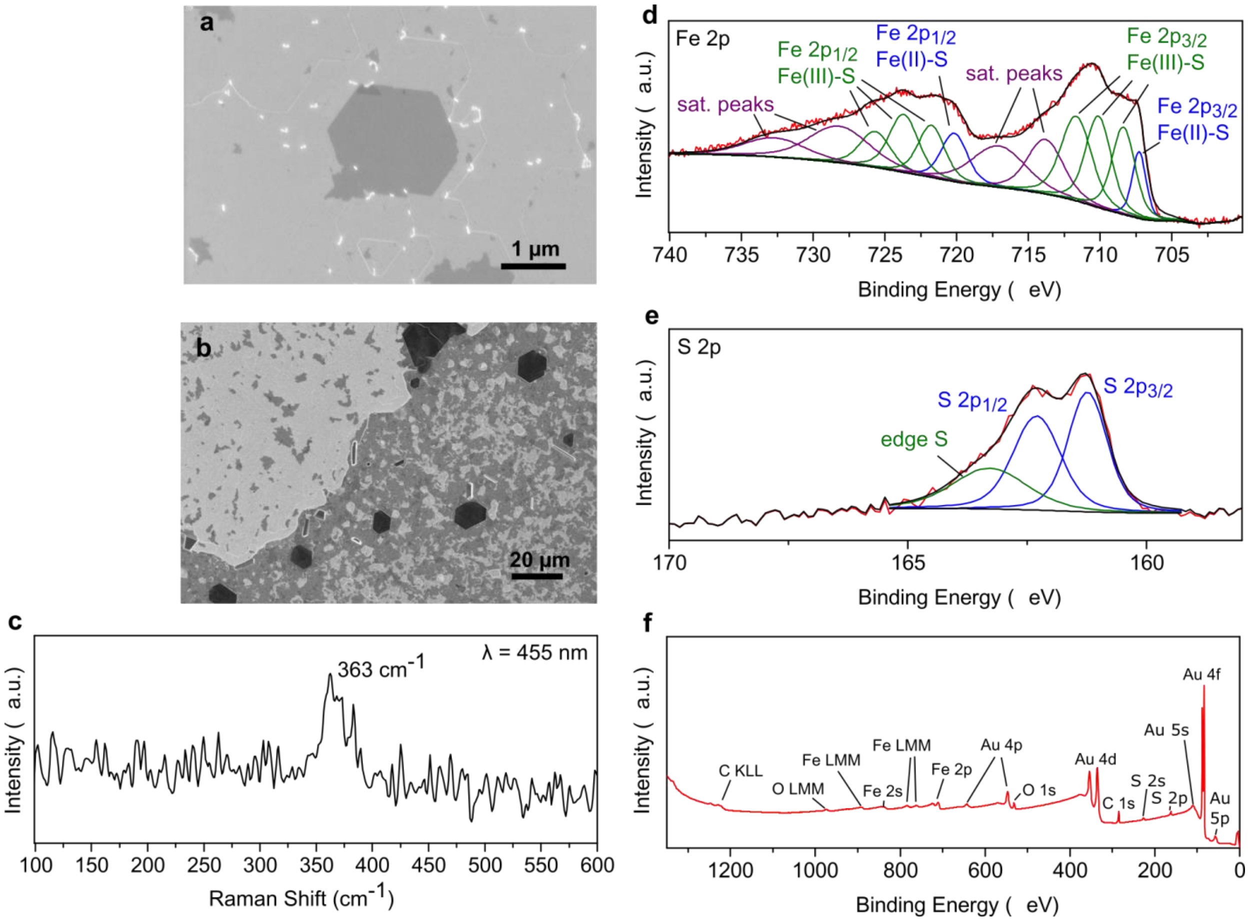

Supplementary Figure 13. Characterisation of iron sulphide (FeS). (a) High magnification and **(b)** low magnification SEM images of FeS on gold. **(c)** Raman spectrum of FeS domains on gold. **(d-f)** XPS spectra of the gold surface post-growth: **(d)** Fe 2p, **(e)** S 2p, and **(f)** survey scan.

Iron sulphides show various growth modes on gold as seen in (b), with some regions on the surface appearing to favour the growth of monolayers, while others favouring the growth of multilayers and 3D crystals. The Fe(II)-S peaks at 707.3 eV and 720.8 eV in the Fe 2p XPS spectrum (d) correspond to reported values for mackinawite[24,25], the layered phase of iron sulphide. We attribute the additional peaks at 708.4 eV, 710.1 eV, 711.7 eV, 722.4 eV, 724.1 eV, and 727.2 eV to spin multiplets of Fe^{3+} oxidation states at the edges and defects in the sulphide domains[25]. The peaks at 713.9 eV, 730.5 eV and 716.9 eV, 733.9 eV are attributed to satellite peaks of the Fe^{2+} and Fe^{3+} oxidation states, respectively. The S 2p region shows 3 peaks at 161.6 eV, 162.9 eV and 164.2 eV, which are attributed to the S $2p_{3/2}$, S $2p_{1/2}$ and edge states of the FeS domains [24,26], respectively. A small amount of oxygen can be seen in the survey spectrum; as such, it is plausible that oxide peaks may be convoluted with the satellite peaks in (d). The Raman spectrum (c) shows a peak at 363 cm^{-1} which is associated with the greigite phase of iron sulphide[27] – it is known that the mackinawite phase oxidizes to the greigite phase upon exposure to ambient air[24,28].

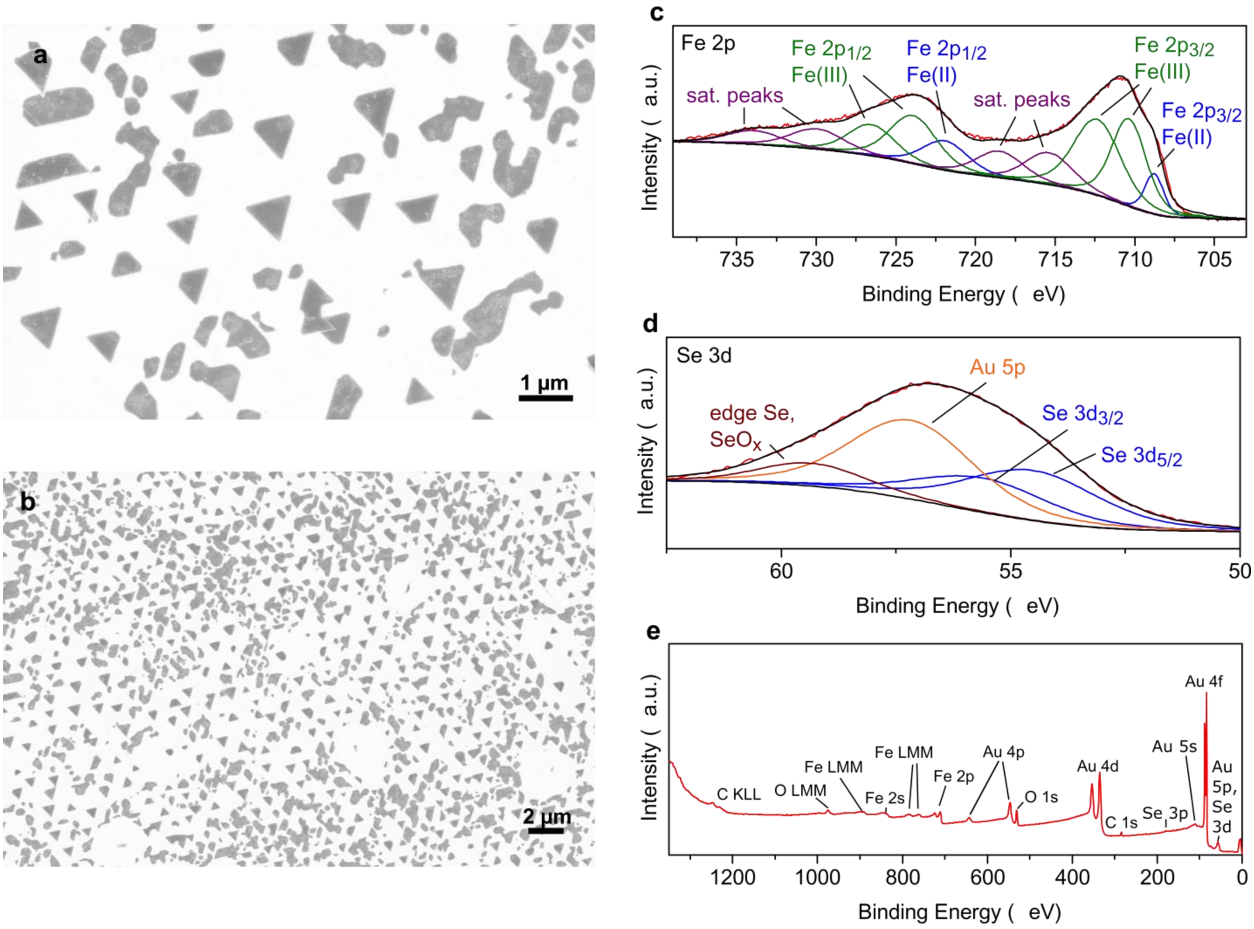

Supplementary Figure 14. Characterisation of iron selenide (FeSe). (a) High magnification and **(b)** low magnification SEM images of FeSe on gold. **(c-e)** XPS spectra of the gold surface post-growth: **(c)** Fe 2p, **(d)** Se 3d, and **(e)** survey scan.

Deconvolution of the Fe 2p XPS spectra for FeSe was done using the FeS spectrum as a guide. Since a considerable amount of oxygen is present from the survey spectrum, we have only identified the peaks from the Fe oxidation states, as the iron oxide peaks are convoluted with the selenide peaks. The Fe(II) peaks at 708.8 eV and 722.1 eV correspond to the Fe^{2+} oxidation state. Additional peaks at 710.4 eV, 712.4 eV, 723.9 eV and 726.5 eV are attributed to Fe^{3+} oxidation states. Satellite peaks at 715.4 eV, 729.9 eV and 718.5 eV, 733.8 eV are attributed to the Fe^{2+} and Fe^{3+} states, respectively. Further oxide/multiplet peaks may be convoluted with the satellite and Fe^{3+} multiplet peaks. The peaks at 54.5 eV and 55.6 eV in the Se 3d region correspond to FeSe[29,30].

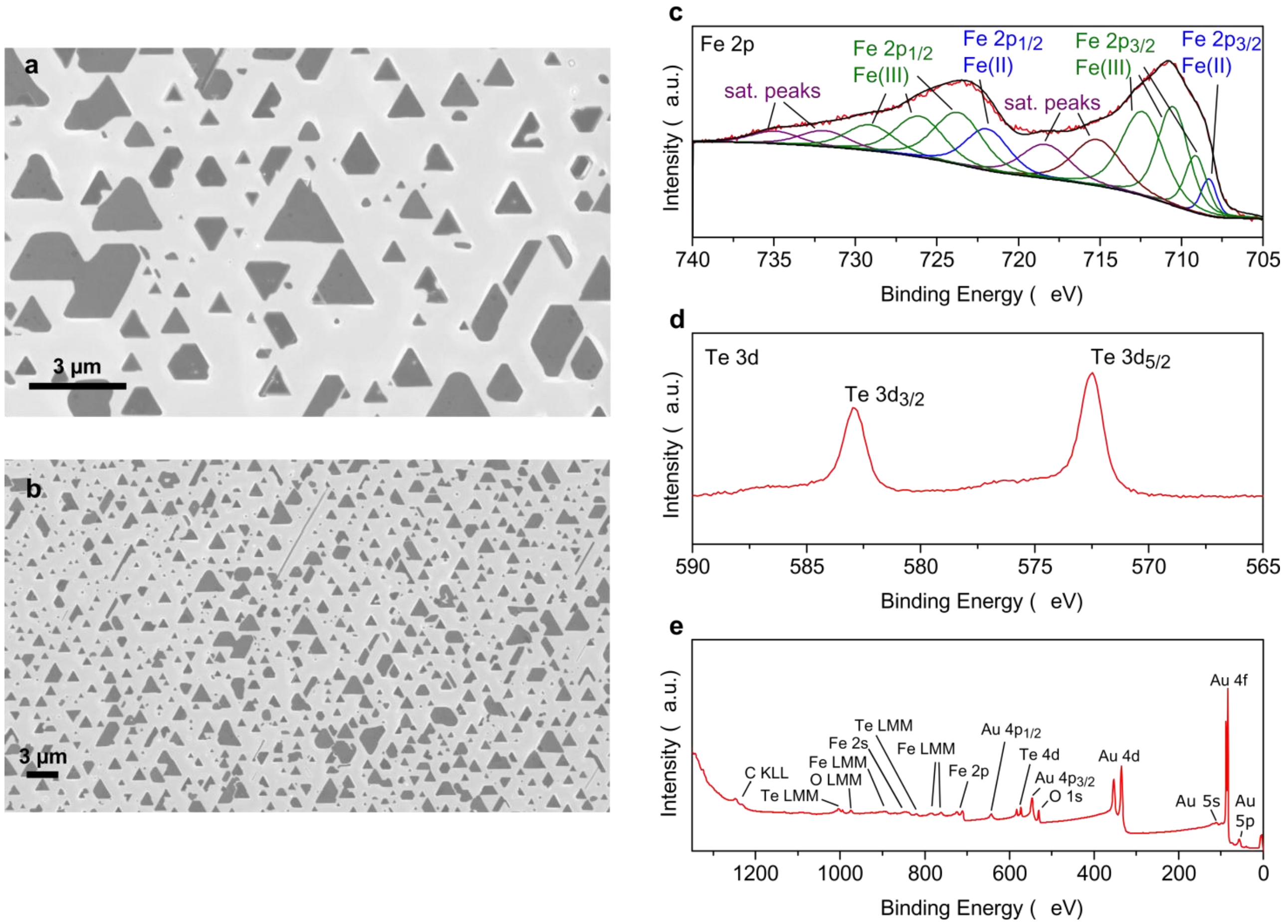

Supplementary Figure 15. Characterisation of iron telluride (FeTe). (a) High magnification and **(b)** low magnification SEM images of FeTe on gold. **(c-e)** XPS spectra of the gold surface post-growth: **(c)** Fe 2p, **(d)** Te 3d, and **(e)** survey scan.

Deconvolution of the Fe 2p region for FeTe was done using FeS and FeSe XPS spectra as guides. As with the case of FeSe, oxygen is present on the surface, and as such only the oxidation states are identified in the Fe 2p region. The peaks at 708.3 eV and 721.4 eV are assigned to the Fe^{2+} oxidation state. Further peaks in (c) at 709.1 eV, 710.5 eV, 712.4 eV, 723.4 eV, 726.1 eV and 729.1 eV are attributed to multiplets of the Fe^{3+} oxidation state. The peaks at 715.2 eV, 731.9 eV and 718.3 eV, 734.9 eV are assigned to the Fe^{2+} and Fe^{3+} satellite peaks, respectively. Te 3d peaks at 572.5 eV and 582.9 eV in (d) correspond to FeTe [11,30]. FeTe also shows evidence of epitaxy with the underlying gold substrate, as seen in the SEM images (a-b).

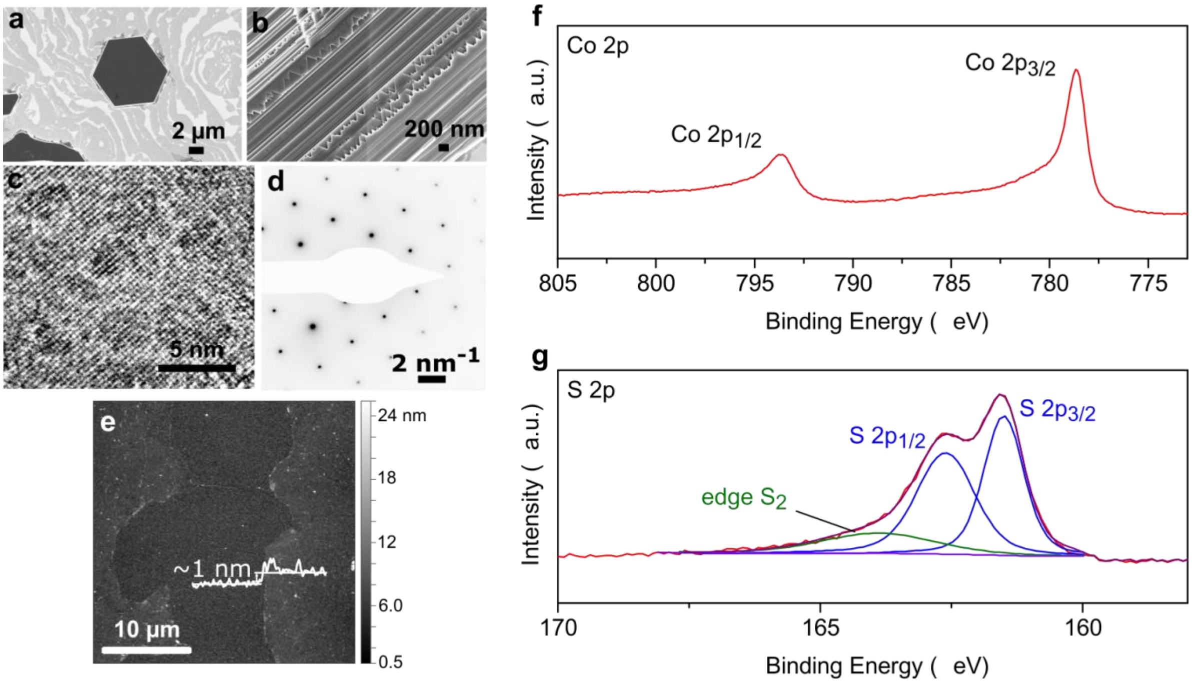

Supplementary Figure 16. Characterisation of cobalt disulphide (CoS_2). (a) Low magnification SEM image of CoS_2 on gold, and **(b)** a high magnification SEM image of one of the edges of the 3D crystals in **(a)**, showing a resemblance to layered crystals. **(c)** high resolution TEM image and (d) SAED pattern of one of the edges of the 3D crystals in (a). Monolayered regions in (a) were too unstable under e-beam irradiation for TEM imaging at 200 kV. (e) AFM of transferred domains in (a) onto 90 nm SiO_2/Si substrate, where the measured thickness of the monolayered regions is ~1 nm, not accounting for chemical contrast between tip and surface. **(f-g)** XPS spectra of the gold surface post-growth: **(f)** Co 2p and **(g)** S 2p regions.

The growth behaviour of sulphides of the ferrous metals Co and Fe bear similarities. We observe monolayer films of CoS_2 (light gray films in (a)) as well as 3D crystals (black hexagon in (a)) similar to those seen for FeS but not seen for any of the other metals tested in this work. The 3D crystals here appear to resemble layered crystals from the SEM (b), but appear to have a cubic crystal structure as seen from the BF-TEM and SAED patterns in (c-d). The XPS Co 2p peaks at 778.7 eV and 793.7 eV, and the S 2p peaks at 161.5 eV and 162.6 eV correspond to literature values for CoS_2[31,32], whereas as the Co:S ratio is 1:2. We attribute the peak at 163.9 eV in the S 2p spectrum to sulphide edges[26]. No Raman signal was measureable on as-grown or transferred films with our setup.

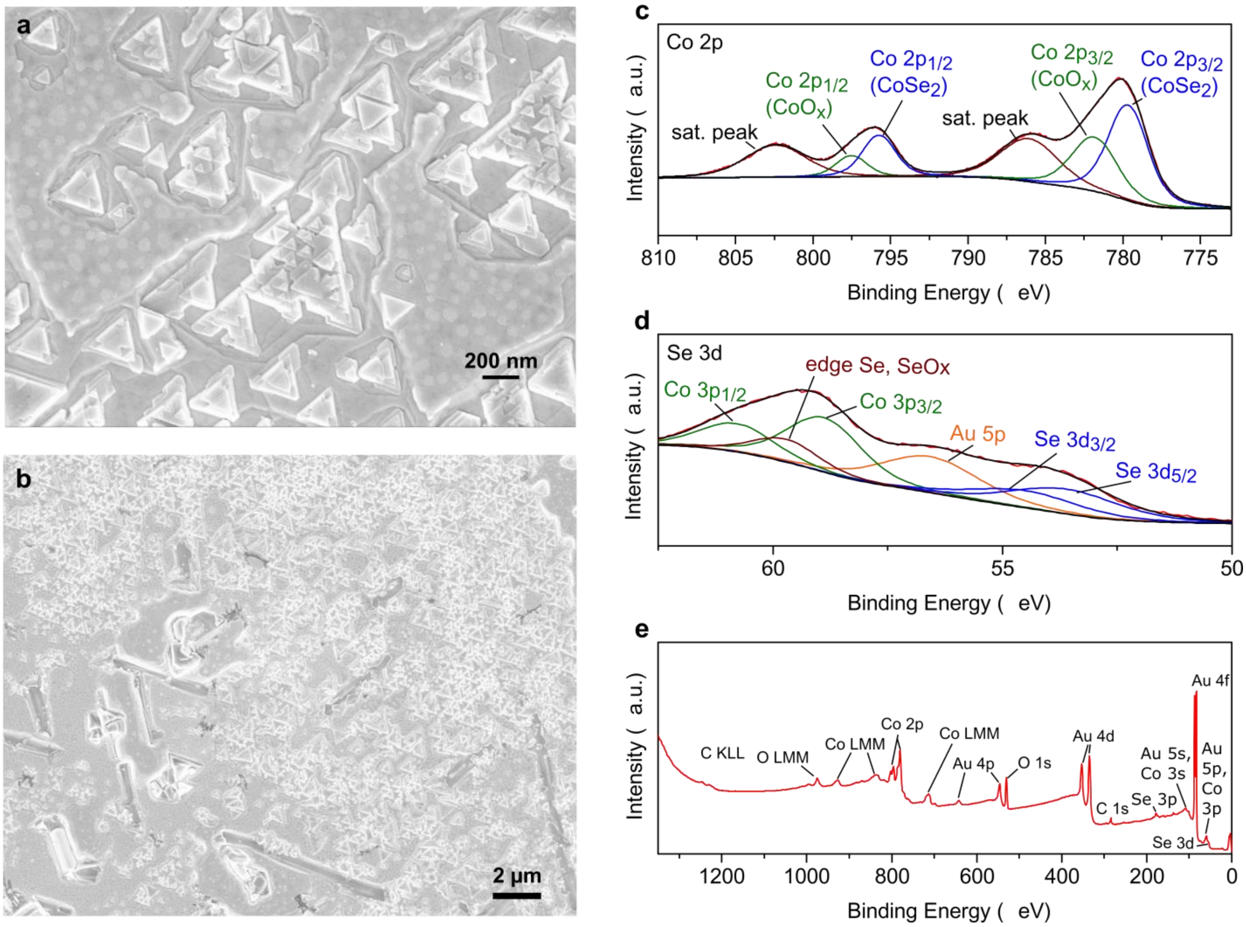

Supplementary Figure 17. Characterisation of cobalt diselenide ($CoSe_2$). (a) High magnification and **(b)** low magnification SEM images of $CoSe_2$ on gold. **(c-e)** XPS spectra of the gold surface post-growth: **(c)** Co 2p, **(d)** Se 3d, and **(e)** survey scan.

A number of structures are observed in the SEM images (a-b), which we believe are a combination of $CoSe_2$ and cobalt oxides. XPS scan of the Co 2p region confirms the presence of $CoSe_2$: Co 2p peaks at 779.7 eV and 795.8 eV correspond to reported values for $CoSe_2$[33,34]. Co 2p peaks at 781.8 eV and 797.5 eV are attributed to cobalt oxides, while the peaks 786.1 eV and 802.4 eV are satellite peaks of the Co^{2+} oxidation state[33,34]. Se 3d peaks at 53.6 eV and 54.5 eV correspond to $CoSe_2$[33–35].

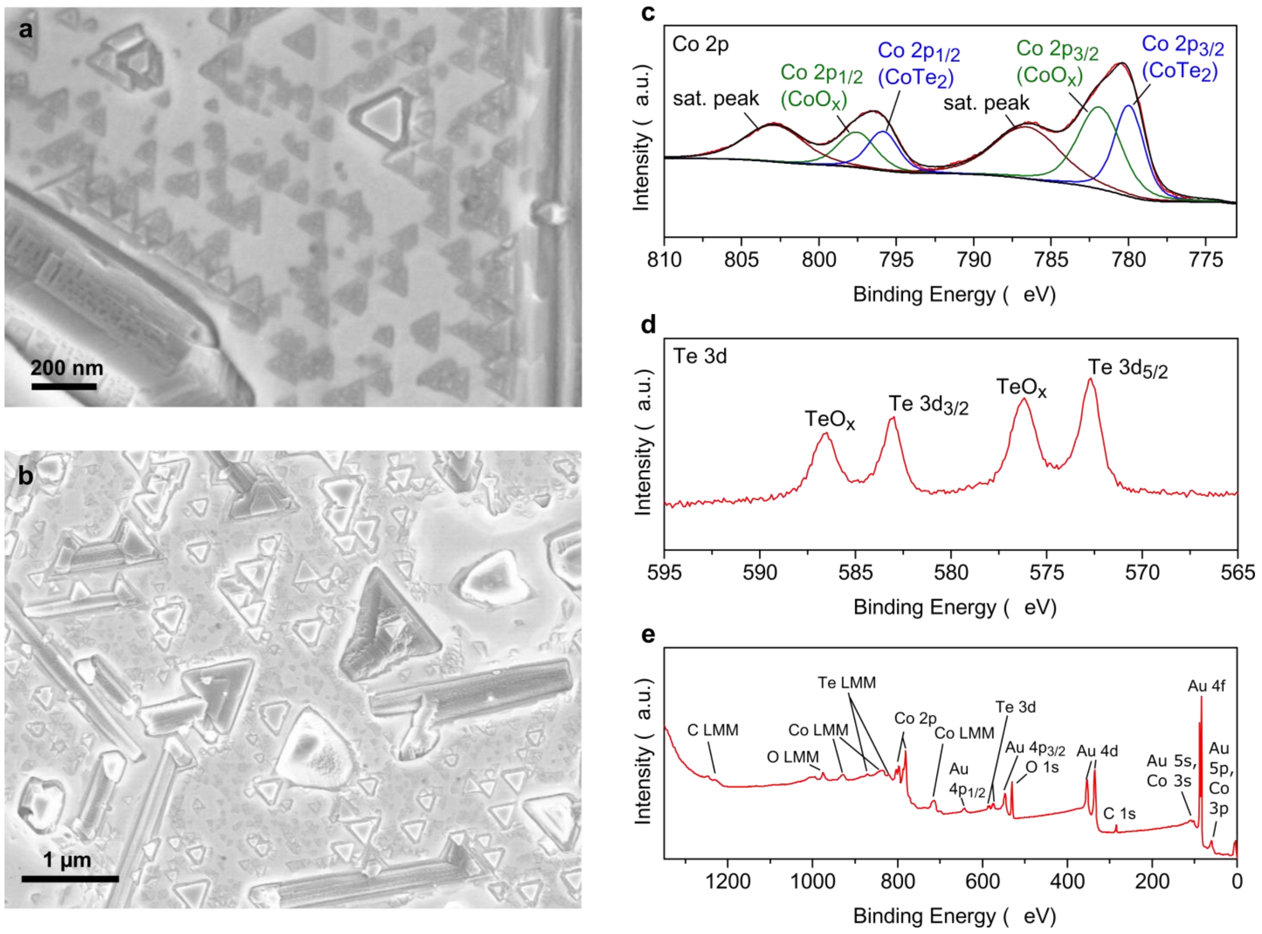

Supplementary Figure 18. Characterisation of cobalt ditelluride ($CoTe_2$). (a) High magnification and **(b)** low magnification SEM images of $CoTe_2$ on gold. **(c-e)** XPS spectra of the gold surface post-growth: **(c)** Co 2p, **(d)** Te 3d, and **(e)** survey scan.

A variety of structures are observed in the SEM images (a-b), which bear resemblance to those seen in the case of $CoSe_2$. Deconvolution of the Co 2p spectrum (c) was done using the $CoSe_2$ case as a guide: Co 2p peaks at 779.9 eV and 795.9 eV correspond to $CoTe_2$, while the peaks at 781.9 eV and 797.6 eV are attributed to cobalt oxides; Peaks at 786.6 eV and 802.9 eV are Co^{2+} satellite peaks. Telluride peaks in the Te 3d region are seen at 572.7 eV and 583.0 eV, while the peaks at 576.2 eV and 586.6 eV correspond to oxidised tellurides[11]. $CoTe_2$ appears to be more air-sensitive than $CoSe_2$, as seen from the oxidised telluride peaks and the prominent cobalt oxide peaks, and from higher oxygen content in the survey spectrum. The triangular structures in (a) correspondingly show visible signs of oxidation/degradation.

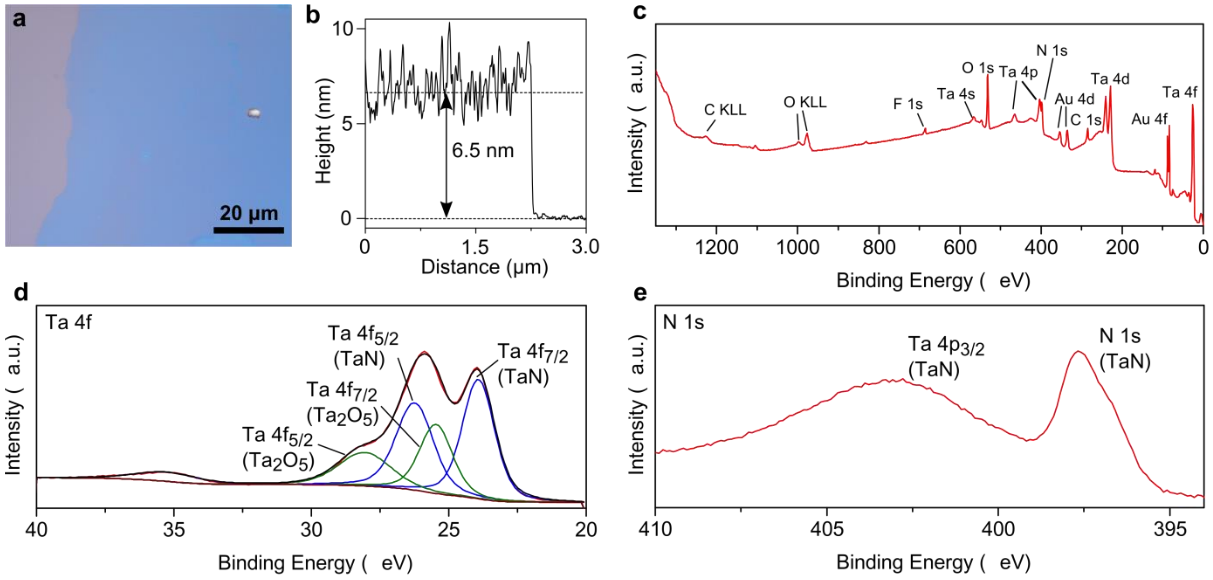

Supplementary Figure 19. Characterisation of ultrathin tantalum nitride films (TaN). **(a)** Optical image of transferred TaN on 90 nm SiO_2/Si substrate. **(b)** AFM line profile of transferred TaN films; the film thickness is ~6.5 nm**. (c-e)** XPS spectra of the gold surface post-growth: **(c)** survey spectrum, **(d)** Ta 4f, and **(e)** N 1s scan. Ta $4f_{7/2}$ and Ta $4f_{5/2}$ peaks at ~24.0 eV and ~26.0 eV in (d) and the Ta $4p_{3/2}$ and N 1s peaks in (e) correspond to reported values for hexagonal TaN[36]; the Ta:N ratio here is ~1.1:1. The films show a noticeable amount of oxidation, as seen by the Ta_2O_5 peaks in (d) and the large oxygen peak in the survey spectrum. The fluorine and carbon signals in the survey spectrum are from backstreamed carbon contaminants[37].

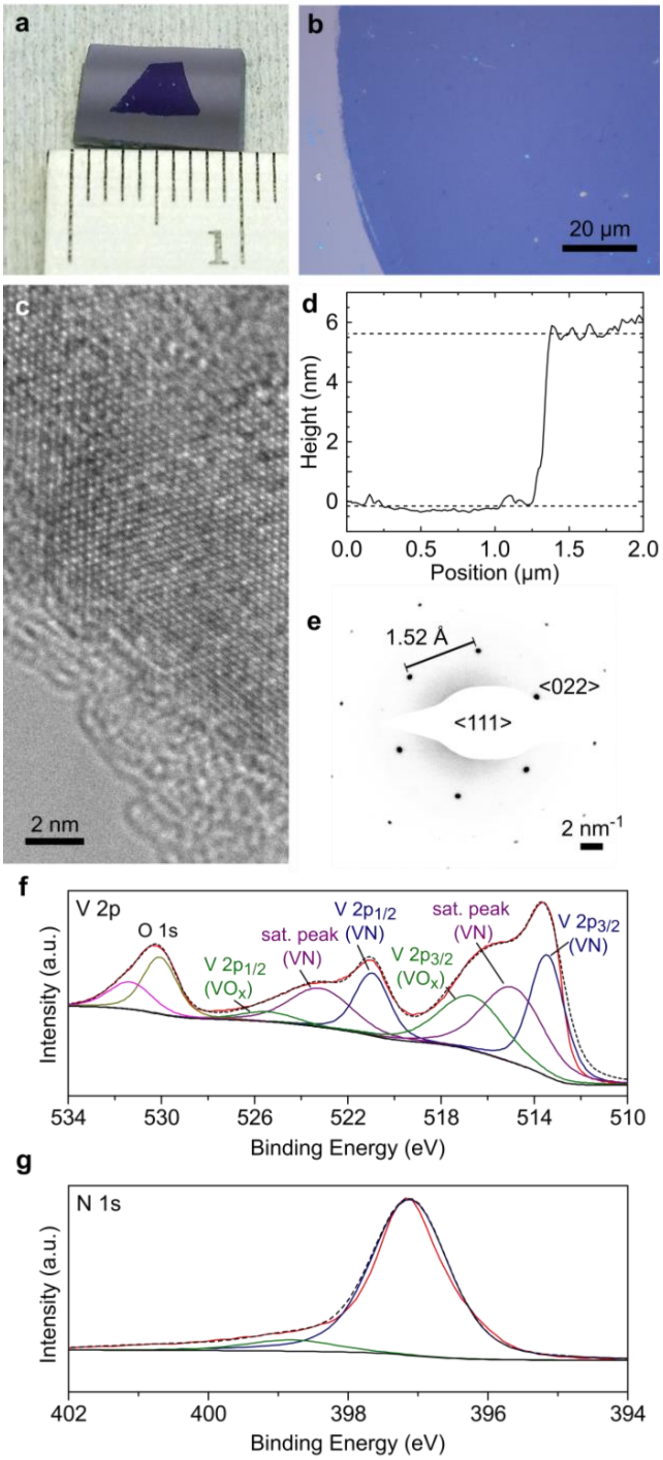

Supplementary Figure 20. Characterisation of ultrathin vanadium nitride films (VN). (a) Photograph of ultrathin vanadium nitride film transferred to 90 nm SiO_2 on Si substrate (scale in cm). **(b)** Optical microscope image of film in (a). **(c)** High resolution bright field TEM micrograph of suspended vanadium nitride film. **(d)** Atomic force microscopy line scan of edge of transferred vanadium nitride film; the measured AFM thickness is ~ 5.5 nm. **(e)** SAED pattern of suspended vanadium nitride film. **(f-g)** XPS spectra of as-grown vanadium nitride on gold. Partial oxidation is visible in the spectrum in (f).

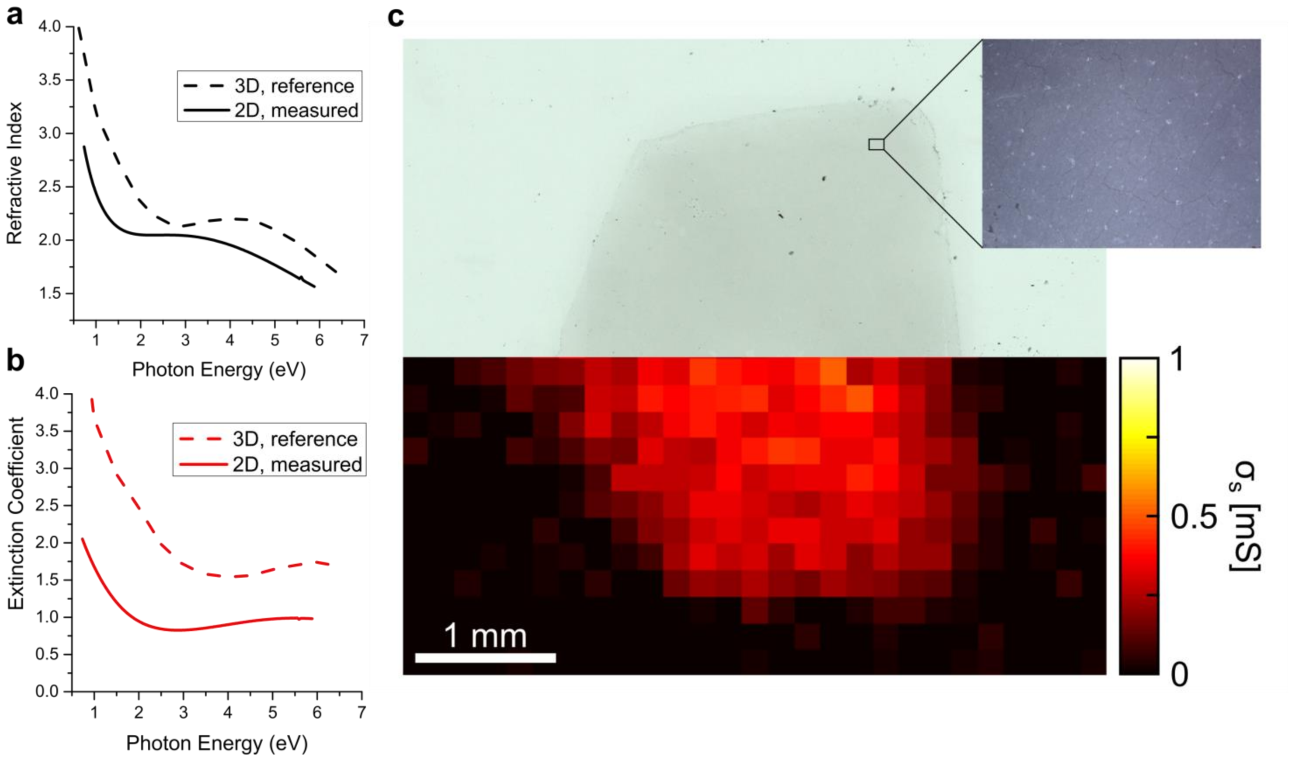

Supplementary Figure 21. Ellipsometry, optical and THz-TDS characterisation of ultrathin vanadium nitride films on quartz. (a-b) Optical functions of 5.5 nm VN (labelled 2D) extracted by least squares fitting of ellipsometry spectra using a parameterised optical function model. The model consists of one Drude oscillator (three fitting parameters) to model free carrier absorption in the infrared, and one Lorentz oscillator (three fitting parameters) to model the response in the visible and near ultraviolet. Optical functions obtained from literature[38] for the bulk VN films are also shown (labelled 3D). **(c)** Optical/THz-TDS sheet conductivity map (mirrored) of transferred VN films on quartz. The sheet conductivity map shows the average sheet conductivity in the range from 0.8-0.9 THz.

Supplementary Figure 20a-b show a centimetre-scale continuous layer of ultrathin vanadium nitride (VN) transferred onto a 90 nm SiO_2 on Si substrate. The size of the film here was limited only by the dimensions of the growth substrate. The thickness of the film is ~5.6 nm, as measured by AFM (Supplementary Figure 20d), and is uniform across the sample, as confirmed by optical microscopy and ellipsometry (Supplementary Figure 20b, Supplementary Figure 21a-b). HRTEM and SAED of the VN films (Supplementary Figure 20c, e) establishes an FCC lattice structure with the {111} facet perpendicular to the growth substrate. Notably, the lattice spacing here, 1.52 Å, is ~4% larger than that of bulk cubic VN, 1.46 Å (ICDD database). XPS characterisation of as-grown VN films on gold (Supplementary Figure 20f, g) shows the expected nitride bonding between vanadium and nitrogen [39], with a V:N ratio of ~1:1. Some degree of oxidation was always present in the

films even when grown under highly reducing conditions, likely due to exposure of the films to ambient conditions. The sheet resistance of transferred VN layers as determined by van der Pauw measurements is ≈ 2 kΩ□$^{-1}$ immediately after transfer, increasing to ≈ 50 kΩ□$^{-1}$ after 100 days of exposure to ambient conditions, which supports the idea that VN layers are air- or humidity-sensitive. Terahertz time domain spectroscopy results performed 15 days after transfer show an average sheet resistivity of 2 kΩ□$^{-1}$ across the transferred VN film (Supplementary Figure 21c).

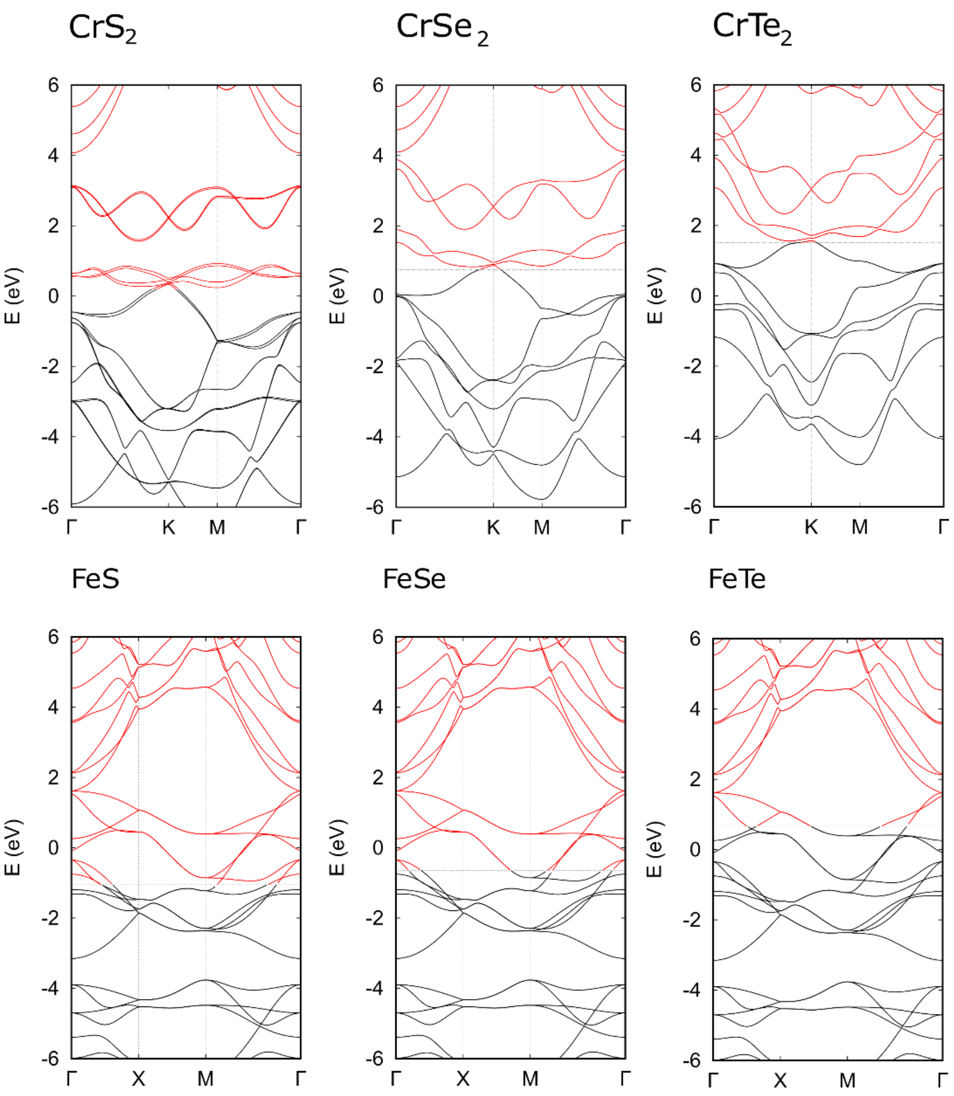

Supplementary Figure 22. Band structure calculations for select materials synthesized. Simulated structures for the CrS_2, $CrSe_2$, and $CrTe_2$ are of the CdI_2 type monolayers, while for FeS, FeSe, FeTe the simulated structure is of the PbO monolayer type.

Supplementary References

1. Buron, J. D. *et al.* Electrically Continuous Graphene from Single Crystal Copper Verified by Terahertz Conductance Spectroscopy and Micro Four-Point Probe. *Nano Lett.* **14**, 6348–6355 (2014).
2. Buron, J. D. *et al.* Graphene Conductance Uniformity Mapping. *Nano Lett.* **12**, 5074–5081 (2012).
3. Kohn, W. & Sham, L. J. Self-consistent equations including exchange and correlation effects. *Phys. Rev.* **140**, A1133–A1138 (1965).
4. Giannozzi, P. *et al.* QUANTUM ESPRESSO: a modular and open-source software project for quantum simulations of materials. *J. Phys. Condens. Matter* **21**, 395502 (2009).
5. Perdew, J. P., Burke, K. & Ernzerhof, M. Generalized Gradient Approximation Made Simple. *Phys. Rev. Lett.* **77**, 3865–3868 (1996).
6. Troullier, N. & Martins, J. L. Efficient pseudopotentials for plane-wave calculations. *Phys. Rev. B* **43**, 1993–2006 (1991).
7. C. J. Shearer, A. D. Slattery, A. J. Stapleton, J. G. Shapter, C. T. Gibson, Accurate thickness measurement of graphene. *Nanotechnology*. **27**, 125704 (2016).
8. K. Godin, C. Cupo, E.-H. Yang, Reduction in step height variation and correcting contrast inversion in dynamic AFM of WS_2 monolayers. *Sci. Rep.* **7**, 17798 (2017).
9. H. R. Gutiérrez *et al.*, Extraordinary room-temperature photoluminescence in triangular WS_2 monolayers. *Nano Lett.* **13**, 3447–3454 (2013).
10. J. F. Moulder, J. Chastain, *Handbook of X-ray photoelectron spectroscopy: a reference book of standard spectra for identification and interpretation of XPS data* (Physical Electronics Division, Perkin-Elmer Corporation, 1992).
11. C. Yim *et al.*, High-performance hybrid electronic devices from layered $PtSe_2$ films grown at low temperature. *ACS Nano*. **10**, 9550–9558 (2016).
12. P. Tonndorf *et al.*, Photoluminescence emission and Raman response of monolayer MoS_2, $MoSe_2$, and WSe_2. *Opt. Express*. **21**, 4908–4912 (2013).
13. Y. C. Jiang, J. Gao, L. Wang, Raman fingerprint for semi-metal WTe_2 evolving from bulk to monolayer. *Sci. Rep.* **6**, 19624 (2016).
14. C.-H. Lee *et al.*, Tungsten Ditelluride: a layered semimetal. *Sci. Rep.* **5**, 10013 (2015).
15. X. Lu *et al.*, Large-area synthesis of monolayer and few-layer $MoSe_2$ films on SiO_2 substrates. *Nano Lett.* **14**, 2419–2425 (2014).
16. W. A. Abdallah, A. E. Nelson, Characterization of $MoSe_2$(0001) and ion-sputtered $MoSe_2$ by XPS. *J. Mater. Sci.* **40**, 2679–2681 (2005).
17. D. Nam, J.-U. Lee, H. Cheong, Excitation energy dependent Raman spectrum of $MoSe_2$. *Sci. Rep.* **5**, 17113 (2015).
18. G. H. Han *et al.*, Absorption dichroism of monolayer 1T′-$MoTe_2$ in visible range. *2D Mater.* **3**, 031010 (2016).
19. S. Zhao *et al.*, Two-dimensional metallic NbS_2: growth, optical identification and transport properties. *2D Mater.* **3**, 025027 (2016).
20. T. Kanazawa *et al.*, Few-layer HfS_2 transistors. *Sci. Rep.* **6**, 22277 (2016).
21. J. Yuan *et al.*, Facile synthesis of single crystal vanadium disulfide nanosheets by chemical vapor deposition for efficient hydrogen evolution reaction. *Adv. Mater.* **27**, 5605–5609 (2015).
22. C. S. Rout *et al.*, Synthesis and characterization of patronite form of vanadium

sulfide on graphitic layer. *J. Am. Chem. Soc.* **135**, 8720–8725 (2013).
23. S. Boursiquot, M. Mullet, M. Abdelmoula, J.-M. Génin, J.-J. Ehrhardt, The dry oxidation of tetragonal FeS_{1-x} mackinawite. *Phys. Chem. Miner.* **28**, 600–611 (2001).
24. D. S. Han, B. Batchelor, A. Abdel-Wahab, XPS analysis of sorption of selenium(IV) and selenium(VI) to mackinawite (FeS). *Environ. Prog. Sustain. Energy*. **32**, 84–93 (2013).
25. A. Bruix *et al.*, *In Situ* detection of active edge sites in single-layer MoS_2 Catalysts. *ACS Nano*. **9**, 9322–9330 (2015).
26. J.-A. Bourdoiseau, M. Jeannin, C. Rémazeilles, R. Sabot, P. Refait, The transformation of mackinawite into greigite studied by Raman spectroscopy. *J. Raman Spectrosc.* **42**, 496–504 (2011).
27. A. R. Lennie, D. J. Vaughan, Spectroscopic studies of iron sulfide formation and phase relations at low temperatures. *Miner. Spectrosc. a Tribut. to Roger G. Burn.* **5**, 117–131 (1996).
28. T. K. Chen *et al.*, Low-temperature fabrication of superconducting FeSe thin films by pulsed laser deposition. *Thin Solid Films*. **519**, 1540–1545 (2010).
29. D. Telesca, Y. Nie, J. I. Budnick, B. O. Wells, B. Sinkovic, Impact of valence states on the superconductivity of iron telluride and iron selenide films with incorporated oxygen. *Phys. Rev. B*. **85**, 214517 (2012).
30. H. Zhang *et al.*, A metallic CoS_2 nanopyramid array grown on 3D carbon fiber paper as an excellent electrocatalyst for hydrogen evolution. *J. Mater. Chem. A*. **3**, 6306–6310 (2015).
31. C.-J. Chen *et al.*, An integrated cobalt disulfide (CoS_2) co-catalyst passivation layer on silicon microwires for photoelectrochemical hydrogen evolution. *J. Mater. Chem. A*. **3**, 23466–23476 (2015).
32. I. H. Kwak *et al.*, $CoSe_2$ and $NiSe_2$ nanocrystals as superior bifunctional catalysts for electrochemical and photoelectrochemical water splitting. *ACS Appl. Mater. Interfaces*. **8**, 5327–5334 (2016).
33. H. Li *et al.*, Template synthesis of $CoSe_2/Co_3Se_4$ nanotubes: tuning of their crystal structures for photovoltaics and hydrogen evolution in alkaline medium. *J. Mater. Chem. A*. **5**, 4513–4526 (2017).
34. C. L. McCarthy, C. A. Downes, E. C. Schueller, K. Abuyen, R. L. Brutchey, Method for the Solution Deposition of Phase-Pure $CoSe_2$ as an Efficient Hydrogen Evolution Reaction Electrocatalyst. *ACS Energy Lett.* **1**, 607–611 (2016).
35. J.-S. M. Lehn, P. van der Heide, Y. Wang, S. Suh, D. M. Hoffman, A new precursor for the chemical vapor deposition of tantalum nitride films. *J. Mater. Chem.* **14**, 3239–3245 (2004).
36. A. Shivayogimath *et al.*, Probing the gas-phase dynamics of graphene chemical vapour deposition using in-situ UV absorption spectroscopy. *Sci. Rep.* **7**, 6183 (2017).
37. J. Pflüger, J. Fink, W. Weber, K. P. Bohnen, G. Crecelius, Dielectric properties of TiC_x, TiN_x, VC_x, and VN_x from 1.5 to 40 eV determined by electron-energy-loss spectroscopy. *Phys. Rev. B*. **30**, 1155–1163 (1984).
38. A. Glaser *et al.*, Oxidation of vanadium nitride and titanium nitride coatings. *Surf. Sci.* **601**, 1153–1159 (2007).
